# Decision Fusion at Pixel Level of Multi-Band Data for Land Cover Classification—A Review

**DOI:** 10.3390/jimaging10010015

**Published:** 2024-01-05

**Authors:** Spiros Papadopoulos, Georgia Koukiou, Vassilis Anastassopoulos

**Affiliations:** Electronics Laboratory, Physics Department, University of Patras, 26504 Patras, Greece; up1055733@ac.upatras.gr (S.P.); gkoukiou@upatras.gr (G.K.)

**Keywords:** land cover classification, decision fusion, pixel-level, multi-band data, thermal images, SAR images

## Abstract

According to existing signatures for various kinds of land cover coming from different spectral bands, i.e., optical, thermal infrared and PolSAR, it is possible to infer about the land cover type having a single decision from each of the spectral bands. Fusing these decisions, it is possible to radically improve the reliability of the decision regarding each pixel, taking into consideration the correlation of the individual decisions of the specific pixel as well as additional information transferred from the pixels’ neighborhood. Different remotely sensed data contribute their own information regarding the characteristics of the materials lying in each separate pixel. Hyperspectral and multispectral images give analytic information regarding the reflectance of each pixel in a very detailed manner. Thermal infrared images give valuable information regarding the temperature of the surface covered by each pixel, which is very important for recording thermal locations in urban regions. Finally, SAR data provide structural and electrical characteristics of each pixel. Combining information from some of these sources further improves the capability for reliable categorization of each pixel. The necessary mathematical background regarding pixel-based classification and decision fusion methods is analytically presented.

## 1. Introduction

In recent years, development in the technology of remote sensing has created remarkable opportunities to use plenty of data sources such as image, spectral, thermal and radar data, among others. This creates a way to exploit valuable information from multi-sensor datasets. Remote sensing comprises information gathering from a long distance using satellites or aircraft, giving the opportunity to observe and measure Earth’s surface features and phenomena. Therefore, this has led to life-changing effects in a lot of scientific areas i.e., agriculture, urban planning, environmental monitoring, natural resource management, change detection and surveillance. To unlock the full potential of these datasets, innovative and efficient feature extraction and classification methods are needed, but we have not achieved satisfactory classification yet. This is where information fusion is used; more specifically, pixel-level decision fusion is used to combine all these characteristics from the desired kind of data in order to achieve a robust approach for land cover classification. During our browsing through the literature, we realized that the segmentation is based on the data used to extract features for a successful decision fusion. The four main categories we have distinguished are hyperspectral (HS), multispectral (MS), synthetic aperture radar (SAR) and optical.

### 1.1. Hyperspectral Data

Hyperspectral data are widely used despite their high dimensionality and complexity because they have proven to be a valuable tool for capturing details about land given that they have numerous narrow contiguous spectral bands with a lot of information. So, two decades ago, Jimenez, Benediksson et al. [1,2,3] proposed a classification method based on decision fusion using majority voting (MV), neural networks and statistical modeling schemes. Later, to add more accuracy, Prasad [4,5] used maximum likelihood estimation (MLE) classifiers with confidence-based weighted MV for hard decisions and linear and logarithmic opinion pools (LOP, LOGP) for soft decisions. In order to achieve better class separation and reduce the impact of trivial spectral variations, in [6,7], support vector machine (SVM) and K-means classifiers are utilized with weighted or unweighted MV. Spectral reflectance and derivative information were explored in [8] using MLE with weighted LOP and MV to fuse the class labels, while in [9], two SVM classifiers were used to achieve an object-based decision fusion. In [10,11,12,13], SVM is the commonly used classifier, but the fusion techniques differ; Ref. [10] presents a composite decision fusion with rule images and two separate kernels; in [11], rule images were used to predict the final class membership of each sample by using classic MV; hard and soft decisions with MV and posterior probability fusion, respectively, were used in [12]; and in [13], naïve Bayes was introduced as a classifier fusion method. In [14,15], Gaussian mixture model (GMM) and Extreme Learning Machine (ELM) classifiers were imported with a multi-classifier decision fusion approach and decision fusion on the probability outputs.

More recent research includes the work of Shokrollahi and Ebadi [16], who improved land cover classification accuracy by using the arithmetic and geometric mean of several decision fusion methods. In [17], the authors introduced a probabilistic fusion approach for pixel-level and superpixel-level hyperspectral image classification using SVM and joint sparse representation (JSR), with the final classification map to be obtained by solving the maximum optimization problem. Furthermore, Ouerghemmi et al. [18] presented a two-step decision fusion strategy using a Gaussian kernel SVM classifier with four classes of rules, fuzzy, Bayesian combination, evidence and margin theory, to be tested. Further, Ref. [19] investigates decision fusion based on multiple features and locality-preserving analysis with GMM and LOGP as a decision fusion scheme. Advancements in feature extraction were also studied in [20,21,22], which incorporated morphological profiles with two classifiers, namely SVM and random forest (RF); joint collaborative representation (JCR) and SVM models; and Gabor features, respectively. Additionally, optimal decision fusion with MV and weighted MV for urban land use and land cover classification using MLE, SVM and multinomial logistic regression (MLR) was explored by Zhong in [23]. Moreover, in [24], the authors introduced a new framework based on probabilistic graphical Markov random field (MRF) and conditional random field (CRF) models.

Furthermore, the development of convolutional neural networks (CNNs) has also influenced decision fusion strategies. In [25], a multi-object CNN model and decision fusion based on fuzzy rules were proposed for coastal wetland classification. In [26], advanced decision fusion data classification was studied using superpixel-level features, RF classifiers and weighted MV as a decision rule. In the work [27], a novel approach called discriminative multiple kernel learning (DMKL) was introduced for spectral image classification. DMKL efficiently learns an optimal combined kernel from basic kernels, enhancing separability in the reproduction kernel Hilbert space. This is achieved by identifying an optimal projective direction using statistical significance, resulting in reduced within-class scatter and increased between-class scatter without the need for exhaustive kernel searches. Experiments on hyperspectral and multispectral datasets show that DMKL outperforms existing methods in both classification performance and computational efficiency for spectral image classification.

### 1.2. Multispectral Data

Multispectral data are also a principal component for land cover classification with a large amount of data across multiple discrete spectral bands. One of the earliest works on decision fusion for multitemporal classification was proposed in [28] using joint likelihood and MV to combine information from different time frames. In [29,30], the authors implemented a method based on statistical and neural network classifiers such as Mahalanobis distance (MD), linear discriminant analysis (LDA), quadratic discriminant analysis (QDA) and conjugate gradient neural network (CGNN) with various voting schemes. In [31], Zhao proposed a new method using SVM and consensus theory that is commonly used in joint reasoning. Generic fuzzy neuron classifiers (FNCs) were explored in [32], which applied a decision fusion technique to combine texture and spectrum features with promising results. Probabilities, possibilities and evidence theories were checked in [33] to achieve a robust multispectral fusion scheme. Recent studies [34,35,36,37] consider that proper decision fusion would be accomplished with the use of SVM, Bayesian networks and naïve Bayesian classifiers, weights of evidence models and decision tree algorithms, respectively. Furthermore, in [38], scene contextual information was exploited for fusion; in [39], SVM and RF classifiers were used with three adaptively weighted decision procedures; in [40], four non-parametric classifiers, namely decision tree (DT), RF, SVM and multilayer perceptron (MLP) were utilized; and in [41], a fuzzy classification with a weighted sum of the membership of imaged objects was implemented in the final classification decision. Besides all these, in [42], SVM and MV were used for decision fusion with a twist; a soft MV had the role of rejecting regions if either the majority or all the classification labels did not agree on one class. In [43,44], MLP-CNN and MRF-CNN classifiers were deployed with decision rules designed primarily based on classification confidence, uncertainty description and classification strategy. As was proposed in [45,46], ML, SVM and RF play their role in feature classification and weighted decisions for the robust combination of individual land cover types. In [47], both pixel- and object-based classification methods were used for the creation of a land use map. In [48], Guan et al. explored a fuzzy classification method using the nearest neighbor (NN) classifier with a weighted decision fusion method. This method adapts the local context based on a context-dependent compromise combination, enhancing the accuracy of the classification process. In [49], a novel method was used for spatiotemporal data fusion with the help of Bayesian decision theory. An object-oriented method for change detection was discussed again in [50] and achieved a solid fusion using fuzzy integral decision rules. In [51], the authors introduced a novel algorithm rooted in the methodology of a fuzzy decision tree, utilizing spectral bands from multispectral imagery as attributes from fuzzy data sources, along with cumulative mutual information for decision tree induction, which not only enhances classification accuracy compared to traditional methods but also achieves substantial data dimensionality reduction through the selection of informative spectral bands. Last but not least, in [52], an SVM classifier, pixel-level layer stacking and Dempster–Shafer theory were used for a vigorous decision fusion for land cover classification with multispectral data.

### 1.3. SAR and Optical Data

Various decomposition methods have been used to extract the biophysical scattering behavior of SAR data and have played a decisive role in the field of target decomposition and image classification. Cloude and Pottier [53] introduced the entropy/anisotropy/alpha (H/A/α) decomposition method, also known as eigenvector–eigenvalue decomposition, which has become a standard tool for characterizing targets and classifying images. Freeman and Durden’s three-component scattering power model [54] is a powerful approach for decomposing POLSAR images under reflection symmetry conditions, utilizing simple physical scattering mechanisms such as surface scattering, double-bounce scattering and volume scattering. Yamaguchi [55] extended this model by including helix scattering power for urban areas and modifying the volume scattering matrix to account for different scattering patterns in vegetated areas. Cameron et al. [56] developed a coherent decomposition method, dividing a polarization scattering matrix into nonreciprocal, maximum symmetric and minimum symmetric scattering components. This approach demonstrates that any group of scatterers within a single-resolution cell can be represented by at most three equivalent scatterers. In addition to these statistics and physical-model-based methods, coherency and covariance matrix decomposition techniques have also made contributions. Krogager [57] presented a new decomposition of complex radar target scattering matrices, particularly on an orthogonal elliptic basis. Van Zyl’s decomposition [58] was introduced for azimuthally symmetrical natural terrain in the monostatic case, offering a general description of the 3 × 3 covariance matrix. Touzi [59] extended the Kennaugh–Huynen coherent target decomposition (CTD) method for decomposing both coherent and partially coherent scattering. These decomposition methods collectively provide valuable tools for understanding and characterizing radar target scattering in various scenarios with SAR and optical data fusion.

From now on, we will refer to classification methods with these two kinds of data, continuing with the work of Yang and Moon [60], who investigated decision fusion using a Bayesian ML classifier and two fusion schemes, namely Dempster’s orthogonal sum and the maximum support rule, for the final land cover types. In [61], an artificial neural network (ANN) was tested for mapping and change detection. An SVM classifier and a cluster of SVM–RF decision rules were proposed in [62]. Cui et al. [63] applied decision fusion to texture features derived from polarimetric data to evaluate levees. Furthermore, in [64], an ML classifier was utilized for the first labeling with MV and qualified majority voting (QMV) as a consensual rule for fusion. Abdikan et al. [65] tested four classifiers, namely SVM, RF, K-nearest neighbor and ML, for the enhancement of land use classification. In [66,67], SVM with correlated probabilities and RF with Dempster–Shafer theory, respectively, were proposed as decision fusion methods using multi-sensor data. Khosravi et al. [68] proposed an improved set of decision trees such as bagged tree (BT), RF, balanced filter-based forest (BFF) and cost-sensitive filter-based Forest (CFF) with an MV rule for decision fusion. Moreover, in [69], researchers explored advanced methods like hierarchical multimodal probabilistic latent semantic analysis (HMpLSA) for land cover fusion. Additionally, in [70], polarimetric SAR and optical data were combined using statistical and decision tree methods. On the contrary, in [71], a fusion classification method was presented based on CNN classifiers and Dempster–Shafer evidence theory. Fuzzy decision fusion schemes for urban remote sensing classification were explored in [72]. In the context of optical data fusion, Cervone and Haack [73] applied three supervised classification machine learning algorithms, namely a decision rule, a decision tree and a Bayesian classifier. On the other hand, moment features from an SVM classifier are exploited [74] and combined using the MV gas decision fusion rule. CNNs were used in both [75,76] to propose state-of-the-art classification approaches with fuzzy rules, Bayesian margin Dempster–Shafer evidence theory and multi-structure joint decision-making strategies. Finally, in [77], Markov random fields were employed for classification with the final land cover labels formed by a Bayesian decision fusion approach.

**Figure 1:**
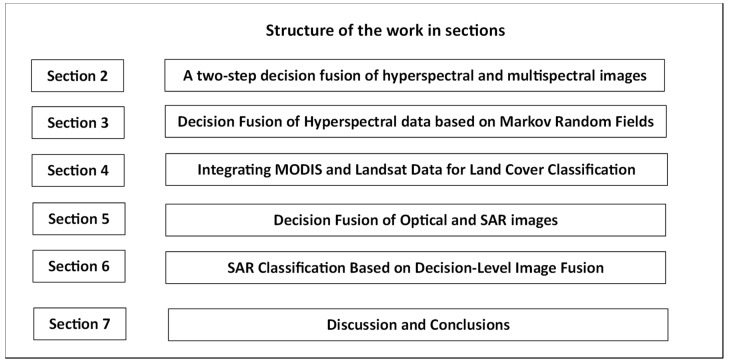
The organization of the present review paper in sections.

In this paper, we present an overview of pixel-based decision fusion methods applied in the domain of remote sensing, highlighting the importance and the resulting possibilities for improving land cover classification accuracy by combining data from multiple sensors and satellites. We selected five papers to present and analyze in our review since they stood out from the body of literature due to their collective significance in addressing critical challenges in remote sensing and land cover classification with decision fusion. Ιn the following sections, as depicted in Figure 1, certain topics of data fusion are covered by the chosen papers. Limited spatial resolution but rich semantic information [18], the robust fusion of more than two decision sources [24], the full exploitation of shape or texture features [48], reducing computational demands of large images using convolution operators [77] and weight-sharing CNN for reducing weights and connections across different layers [71]. Analytically, Ouerghemmi, Le Bris et al. [18] focused on resolving the spatial and spectral resolution tradeoff in remote sensing by proposing a two-step fusion method that combines multispectral and hyperspectral imagery. This approach is extended to sensors with limited spatial resolution while decision-level fusion is emphasized. The global regularization framework enhances classification by considering spatial relationships and image contrast. The paper provides a comprehensive methodology for urban classification, making it a valuable contribution. Furthermore, Andrejchenko et al. [24] introduced hyperspectral image classification using the innovative method of Markov and conditional random fields for decision fusion. Their work combines fractional abundances and class probabilities, enhancing pixel characterization. The application of these fields offers a unique perspective and can lead to improved results in this underexplored area of research. In the paper [48], Guan et al. introduced a multilevel decision fusion scheme for combining Moderate Resolution Imaging Spectroradiometer (MODIS) and Landsat data, promising more accurate land cover classification. This approach is especially beneficial in regions with challenging weather conditions, such as cloud cover and rainfall, where traditional high-resolution data may be less effective. Additionally, Maggiolo, Solarna et al. [77] addressed the crucial topic of climate change monitoring by combining optical and SAR sensors. Decision fusion is employed to combine probabilistic decisions from these sources, considering their unique characteristics and associated uncertainties. This approach enhances the quality of land cover mapping by leveraging the complementary strengths of each sensor type. Finally, in the paper [71], Zhu, Pan et al. introduced a novel decision fusion technique using the Dempster–Shafer theory to combine classification results from multi-band SAR images. It addresses the challenge of uncertainty in classification outcomes, which is vital in remote sensing. The employment of evidence theory for fusion is innovative and provides a structured framework for robust decision making, particularly in applications like disaster management and agriculture. These papers collectively enrich our understanding of data fusion in remote sensing and its broader implications for various domains.

## 2. A Two-Step Decision Fusion of Hyperspectral and Multispectral Images for Urban Classification [18]

In order to map urban environments successfully, is necessary to use images with extremely high spatial resolution, typically less than 5 m. However, high-spatial-resolution sensors often come with limits in the configuration of their spectrum, typically comprising just three or four bands like RGB or RGB-NIR, which restricts their capacity to distinguish between fine-grained classes [78,79,80] and hampers classification accuracy when compared to multispectral or hyperspectral sensors. Unfortunately, the spatial resolution of the latter sensors is generally lower. To address the imperfections of both types of sensors, the integration of multispectral and hyperspectral imagery could be a viable solution, given the ability to exploit their complementary characteristics. This integration aims to provide two key benefits: (1) plenty of geometrical and textural details to finely delineate objects and (2) a plethora of spectral information to effectively differentiate between various classes. Consequently, the fusion of these sensor types should improve the classification performance while maintaining the peak spatial resolution. 

The fusion process can be conducted at three separate levels:

1. At the observation level: This involves the combination of a high-resolution panchromatic (PAN) image with a lower-resolution multispectral image to generate a high-resolution multispectral image. A comprehensive overview of these types of methods can be found in reference [81].
2. At the feature level: As described in references [82,83,84], this level entails the application of a single classification approach utilizing features extracted from both data sources.
3. At the decision level: As detailed in references [72,85], this level involves the integration of various classification maps derived from diverse datasets.

The research outlined in [18] focuses on developing a versatile fusion method that has applicability beyond hyperspectral/multispectral data, encompassing sensors with limited spatial resolution but rich semantic information, as well as sensors with very high spatial multispectral capabilities. This paper focuses on fusion at the decision level. The main goal of this method is to address both semantic and spatial uncertainties, and it consists of two key stages: decision fusion at the pixel level and classification optimization through a global regularization framework. Several decision fusion techniques are explored, including fuzzy, Bayesian, margin-based and Dempster–Shafer-based rules. The fusion process is further refined in the second stage using a graph-cut algorithm that incorporates a spatial regularization term sensitive to image contrast.

**Figure 2:**
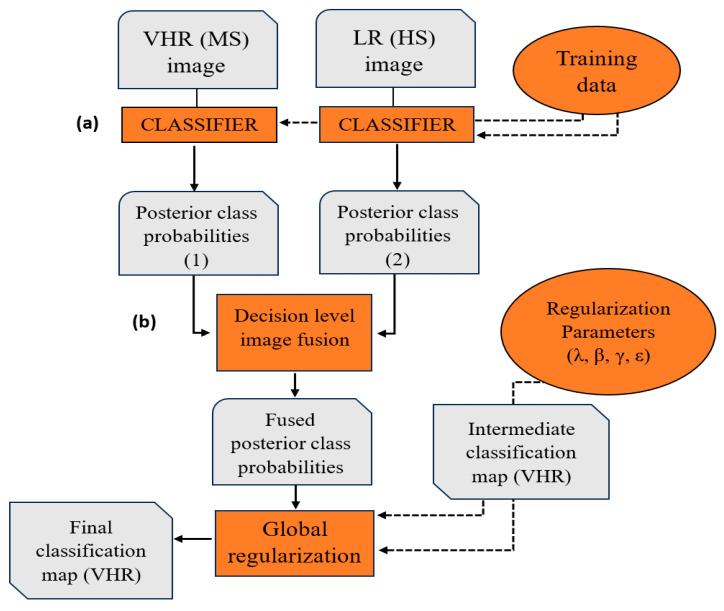
Multi-source data fusion in two steps. (**a**) Classification and (**b**) Decision Fusion.

The method can be broken down into three main steps: (a) classification of hyperspectral and multispectral images and generation of posterior probabilities; (b) fusion of these posterior probabilities at the decision level; (c) classification optimization (Figure 2). A Gaussian kernel SVM classifier [86] is employed in the first step, followed by decision fusion applied to the resulting posterior class probability maps. The last step involves a global regularization of the classification map obtained in the second step, implemented using a graphical model with fit-to-data and contrast-sensitive regularization terms.

The decision fusion rules employed in this study exclusively rely on class membership probabilities created from the classifier at the pixel level. The fusion process is executed on a pixel-by-pixel basis, combining class membership probabilities from each data source. For this research, ten different decision rules were tested.

### 2.1. Fuzzy Rules

**Theoretical approach and general characteristics:** If we consider a reference set L of classes, a set A in L containing ordered pairs is a fuzzy set:

(1) $$A=\left[\left(x,P_{A}\left(x\right)|x\in L\right)\right]$$

where $P_{A}∶L\to [0,\;1]$ is referred to as the membership probability of A in L. This function is characterized by having a finite supremum. The intersection between two fuzzy sets $P_{A}\;and\;P_{B}$ is determined by taking the minimum of their respective membership probabilities:

(2) $$\forall x\in L\left(P_{A\;}\cap \;P_{B}\;\right)\left(x\right)=Min\left(P_{A}\left(x\right),P_{B}\left(x\right)\right).$$

The maximum of the following expression provides the union of the two fuzzy sets $P_{A\;}and\;P_{B}$:

(3) $$\forall x\in L\left(P_{A\;}\cup \;P_{B}\;\right)\left(x\right)=Max\left(P_{A}\left(x\right),P_{B}\left(x\right)\right).$$

The next expression provides the complement of a fuzzy set *P_A_*:

(4) $$\forall x\in LP_{\bar{A}}\left(x\right)=1-P_{A}\left(x\right)$$

**Quantification of discordance between two sources:** Let us examine two sources, A and B, along with their associated probabilities, $P_{A\;}and\;P_{B}$. To quantify the disagreement between these sources, the Dubois and Prade measure (1−K) [87] is employed, where

(5) $$K={Sup}_{x}Min\left(P_{A}\left(x\right),P_{B}\left(x\right)\right)$$

**Confidence measure (level of confidence):** To mitigate the impact of untrustworthy data within each fuzzy set, a weighting factor denoted as $w_{i}$ is introduced, as proposed in [72]. Let us consider the fuzzy set $P_{i}\left(x\right)$, where i represents the number of source or classification images and x represents a pixel belonging to source i. In a rational sense, a classifier is deemed reliable when for a given pixel x, one class exhibits a high membership while the memberships of other classes are low. On the contrary, if more than one class shows a high membership, the fuzzy set will exhibit a high degree of fuzziness, signifying that the classifier’s reliability regarding pixel x is reduced. Building upon this premise, each fuzzy set can be weighted by $w_{i}$ to diminish the influence of unreliable information. This can be expressed as follows:

(6) $$w_{i}=\frac{\sum_{k=0,k\neq i}^{n}H_{aQE}\left(P_{k}\right)}{\left(n-1\right)\sum_{k=0}^{n}H_{aQE}\left(P_{k}\right)}$$

where n is the number of sources and $H_{aQE}\left(P_{k}\right)$ the fuzziness degree of source k. $H_{aQE}$ is a measure of fuzziness which is called the a-quadratic entropy (QE) [88].

**Fuzzy rules:**

Five fuzzy operators are investigated for fusion:

(1) A conjunctive T-norm Min operator:
  (7) $$P_{fusion}\left(x\right)=Min\left(P_{A}\left(x\right),P_{B}\left(x\right)\right).$$
(2) A disjunctive T-norm Max operator:
  (8) $$P_{fusion}\left(x\right)=Max\left(P_{A}\left(x\right),P_{B}\left(x\right)\right).$$
(3) A compromise operator [89]:
  (9) $$P_{fusion}\left(x\right)=\left\{\begin{array}{cc} Max\left(T_{1},Min\left(T_{2},\left(1-K\right)\right)\right) & if\;\left(1-K\right)\neq 0\;\\ Max\left(P_{A}\left(x\right),P_{B}\left(x\right)\right) & if\;\left(1-K\right)=1 \end{array}\right\}$$

where T1=MinPAx,PBxK, $T_{2}=Max\left(P_{A}\left(x\right),P_{B}\left(x\right)\right).$

- When the dissension between A and B is low (i.e., $\left(1-K\right)\approx 0$), the operator action is conjunctive.
- When the dissention between A and B is high (i.e., $\left(1-K\right)\approx 1$), the operator action is disjunctive.
- When the dissention is partial (i.e., $0<\left(1-K\right)<1$), the operator acts in a compromise way.

(4) Operator [87] rules in priority are considered:
  (10) $$P_{fusion}\left(x\right)=Max\left(P_{A}\left(x\right),Min\left(P_{B}\left(x\right),K\right)\right).$$
  (11) $$P_{fusion}\left(x\right)=Min\left(P_{A}\left(x\right),Max\left(P_{B}\left(x\right),\left(1-K\right)\right)\right)$$

For a large value of the conflict between A and B, for both operators, (i.e., K≈0), $P_{A}$ contradicts $P_{B}$, and only $P_{A}$ is considered, while $P_{B}$ is considered as a distinct sample of information.

(5) An *accuracy-dependent (AD)* operator [72] takes into account local and global confidence measurements:
  (12) $$P_{fusion}^{j}\left(x\right)=Max\left(Min\left(w_{i}P_{i}^{j}\left(x\right),f_{i}^{j}\left(x\right)\right),\;i\in \;\left[1,n\right]\right)$$

where $f_{i}^{j}$ represents the overall source’s i level of confidence with respect to class j, $P_{i}^{j}$ denotes the class membership information from source i and $w_{i}$ serves as a normalization factor. This operator’s role is to certify that for each class, only reliable sources are considered, based on the prearranged coefficients $f_{i}^{j}$ for assessing reliability.

### 2.2. Bayesian Combination

Combinations of basic Bayesian sum and product membership probabilities are utilized. In this approach, each membership probability is multiplied by a pointwise measure. This enables the assessment of how these operators compare to more intricate combinations. The fusion process involves employing Bayesian sum and product operators in the following manner:

(13) $$P_{fusion}\left(x\right)=P_{A}\left(x\right)+P_{B}\left(x\right).$$

(14) $$P_{fusion}\left(x\right)=P_{A}\left(x\right).P_{B}\left(x\right).$$

### 2.3. Margin-Based Rule (Margin-Max)

Now, consider two sources, A and B, where $S=\left\{A,B\right\}$, and a set of different classes $L={\left\{c_{i}\right\}}_{i\in [1,n]}$. Let $P_{s}^{\left(c\right)}\left(x\right)$ represent the pointwise membership probability of pixel x in a class c, based on source s. The margin of source s at pixel x is

(15) $${margin}^{\left(s\right)}\left(x\right)=P_{s}^{\left(cbest1\right)}\left(x\right)-P_{s}^{\left(cbest2\right)}\left(x\right)$$

where $cbest1={argmax}_{c\in L}P_{s}^{\left(c\right)}\left(x\right)$ and $cbest2={argmax}_{c\in L\backslash cbest1}P_{s}^{\left(c\right)}\left(x\right)$.

In this study, we investigate the Max-Margin fusion method to compute the combined membership probabilities of two sources, A and B, where $S=\left\{A,B\right\}$, and a set of distinct classes $L={\left\{c_{i}\right\}}_{i\in [1,n]}$. ∀x,∀c∈L,

(16) $$P_{fusion}^{\left(c\right)}\left(x\right)=P_{S_{best}}^{\left(c\right)}\left(x\right)$$

where $S_{best}={argmax}_{S\in C}{margin}^{\left(s\right)}\left(x\right)$

### 2.4. Dempster–Shafer Evidence Theory-Based Rule

The data coming from a source s for a specific class c are expressed using a mass function $m_{c}|m_{c}\;\in \;\left[0,\;1\right]$ based on the Dempster–Shafer (DS) theory. The restriction on these composite classes is that they can contain, at most, two different classes concurrently. Next, the masses are divided into each of these classes as follows:

- $m\left(\emptyset \right)=0$
- Simple classes: ∀c∈L, ∀pixel x, and ∀s∈S, $m_{s}^{\left(c\right)}\left(x\right)=P_{s}^{\left(c\right)}\left(x\right)$, where m is the mass affected in class c by source s, and *P* is a pointwise membership probability of the considered class.
- Compound classes: The compound class masses are here generated as follows: ∀c1,c2∈L, ∀pixel x and ∀s∈S.

(17) $$m_{s}^{\left(c1\cup c2\right)}\left(x\right)=\left(P_{s}^{\left(c1\right)}\left(x\right)+P_{s}^{\left(c2\right)}\left(x\right)\right)\times \left(1-Max\left(P_{s}^{\left(c1\right)}\left(x\right),\;P_{s}^{\left(c2\right)}\left(x\right)\right)\right)+Min\left(P_{s}^{\left(c1\right)}\left(x\right),\;P_{s}^{\left(c2\right)}\left(x\right)\right).$$

Normalization is as follows: $\sum_{c\in L}^{\left(s\right)}$,$m_{s}^{\left(c\right)}\left(x\right)=1$. The *DS* conflict measure between two sources, A and B, is

(18) $$K\left(x\right)=\sum_{\begin{array}{c} c,d\in \overset{´}{L}\\ c\cap d=\emptyset \end{array}}^{\left(s\right)}m_{A}^{\left(c1\right)}\left(x\right)m_{B}^{\left(d\right)}\left(x\right)$$

where $c,d\in \overset{´}{L}$, are compound classes with c∩d=∅.

The probability masses are finally merged:

(19) $$m_{fusion}^{\left(c\right)}\left(x\right)=\frac{1}{1-k\left(x\right)}\sum_{c1,c2\in \overset{´}{L}}^{\left(s\right)}m_{A}^{\left(c1\right)}\left(x\right)m_{B}^{\left(c2\right)}\left(x\right),$$

### 2.5. Global Regularization

The global regularization model is used in order to improve the final performance of the classification fusion. The problem is presented using a graphical model of energy character and is solved as a minimum cut problem, as shown in reference [90].

**Model definition:** The energy term consists of two components: $E_{data}$, which handles data-related aspects, and $E_{reg}$, which addresses regularization. In adapting the model described in [91], it was tailored specifically for classification rectification rather than fusion. This model leverages a graphical framework, where the energy model is a probabilistic function reliant on the posterior probability $P_{fusion}$. For a given classification map C, the energy term can be written as follows:

(20) $$E\left(P_{fusion},C_{fusion},C\right)=\sum_{x\in I_{MS}}E_{data}\left(C\left(x\right)\right)+\lambda \sum_{\begin{array}{c} x,y\in N\\ x\neq y \end{array}}E_{reg}\left(C\left(x\right),C\left(y\right)\right)$$

where

$E_{data}\left(C\left(x\right)\right)=f\left(P_{fusion}\left(C\left(x\right)\right)\right)$, $E_{reg}\left(C\left(x\right)=C\left(y\right)\right)=f\left(P_{fusion}\left(C\left(x\right)\right),C_{fusion}\right)$, $E_{reg}\left(C\left(x\right)\neq C\left(y\right)\right)=h\left(P_{fusion}\left(C\left(x\right)\right),C_{fusion}\right)$.

λ ∈ [0, ∞] is a parameter comprising data and regularization terms, *N* is the eight connexity neighbors.

$E_{data}$ is a fit-to-data attachment term which is a function of the probability map $P_{fusion}$ which models the result of the classification fusion, defined by the function f:

(21) $$f\left(t\right)=-\log\left(t\right)\;with\;t\in \left[0,1\right].$$

The role of the function f is to ensure that when the probability of a pixel x belonging to class $C\left(x\right)$ is close to 1, the $E_{data}$ remains minimal and has little effect on the total energy E. Conversely, if the probability of a pixel x belonging to the class $C\left(x\right)$ is low, $E_{data}$ approaches its maximum value, thus disallowing such a configuration. Meanwhile, $E_{reg}$ represents a regularization term that characterizes the interactions between a pixel x and its eight neighboring pixels.

A slightly improved Potts model is also used. MS image $I_{MS}$ contrast information [83] is integrated using the model and verifying the following:

(22) $$E_{reg}\left(C\left(x\right)=C\left(y\right)\right)=0,\;E_{reg}\left(C\left(x\right)\neq C\left(y\right)\right)=\left(1-\gamma \right)\left(1-P_{fusion}{\left(C_{fusion}\left(x\right)\right)}^{\beta }\right)+\gamma \;V\left(x,y,\epsilon \right),$$

where β∈ [0, ∞] serves as a tradeoff parameter that balances the influence of the smoothing criterion with the significance of $C_{fusion}$ within the model. Additionally, *V* represents a measure of contrast, γ determines the tradeoff between the foundational model guided by the decision fusion classification $C_{fusion}$ and the incorporated contrast term $V\left(x,y,\epsilon \right)$ and *ϵ* is a parameter that modifies the standard deviation in the exponential term. The contrast term, as described in reference [92], can be expressed as follows:

(23) $$V\left(x,y,\epsilon \right)=\frac{1}{n}\sum_{i\in \left[0,dim\right]}V_{i}{\left(x,y\right)}^{\epsilon }\;with\;\epsilon \;\in \;\left[0,\infty \right]$$

where $V_{i}\left(x,y\right)=exp\left(\frac{-{\left(I_{i}\left(x\right)-I_{i}\left(y\right)\right)}^{2}}{{2\left(I_{i}\left(x\right)-I_{i}\left(y\right)\right)}^{2}}\right)$, n is the dimension of image $I_{MS}$ and $I_{i}$ is the intensity for pixel x in the MS image.

The restructured Potts model for the regularization term offers a more effective approach to the smoothing process. Specifically, when $C\left(x\right)\neq C\left(y\right)$, $E_{reg}\left(C\left(x\right)\neq C\left(y\right)\right)$ becomes a function influenced by both $P_{fusion}$ and V. If $P_{fusion}\left(C_{fusion}\left(x\right)\right)$ approaches 1, it implies that decision fusion assigns a high level of confidence to pixel x belonging to class $C_{fusion}$. In such cases, $E_{reg}$ predominantly relies on V to determine whether the configuration $C_{fusion}$ is preferred or not. Conversely, when $P_{fusion}\left(C_{fusion}\left(x\right)\right)$ approaches zero, $E_{reg}$ is elevated, signaling that the configuration *C_fusion_* is more likely to be rejected.

**Parameter setup:** Within the energy term E (20), we utilize four key parameters that govern the extent of regularization: λ, γ, β and ϵ. λ ∈ [0, ∞] acts as a tradeoff parameter determining the balance between the contributions of the terms $E_{data}$ and $E_{reg}$. Increasing λ intensifies the regularization effect. γ ∈ [0,1] serves as a tradeoff parameter that governs the equilibrium between the fundamental energy model and the refined model that incorporates the contrast measure. Lastly, ϵ ∈ [0, ∞] functions as a parameter that influences the impact of the contrast measure within the energy term. In the context of a Potts model, these parameters are configured as follows:

$$\gamma =0\;and\;\beta \to +\infty \;\left(or\right)\;\gamma =1\;and\;\beta \to +\infty \;and\;\epsilon =0$$

In conclusion, this study presents a two-step approach that addresses the fusion of multisource data and global regularization. The ultimate phase involves optimizing the results of decision fusion through global regularization to enhance classification. The core concept revolves around regularization applied to individual pixel memberships and their spatial relationships, as well as considering an image contrast measure when evaluating neighboring pixels.

## 3. Decision Fusion of Hyperspectral Data Based on Markov and Conditional Random Fields [24]

In recent years, hyperspectral image classification has garnered significant attention in research due to the wealth of spectral information present in hyperspectral images (HSIs). On the other hand, in the realm of remote sensing, obtaining ground truth information is a challenging and costly process, typically resulting in a restricted pool of training data. Coupled with the high number of spectral bands, this gives rise to the Hughes phenomenon [93], making HSI classification a formidable task. Furthermore, the substantial spectral similarity among certain materials adds complexity, ambiguity and intricacy to the classification problem. Additionally, the relatively low spatial resolution of HSIs leads to a significant number of mixed pixels, further complicating the classification task.

To address these challenges, researchers have pursued a more comprehensive characterization of pixels and their local context. Many spatial–spectral methods have been developed to incorporate spatial information through contextual features. Typically, spatial–spectral methods utilize feature vectors with significantly higher dimensionality compared to spectral-only methods. This discrepancy can diminish the classifiers’ capacity to generalize effectively with a consistent volume of training data. To address this challenge, feature fusion and decision fusion methods have arisen. In feature fusion, the features are directly amalgamated, frequently through a stacked architecture or by employing composite or multiple kernels.

Decision fusion methods acquire probability values (decisions) from distinct individual feature sets using probabilistic classifiers and subsequently fusing these decisions. Several studies have employed decision fusion rules for the combination of pixel-based classification outcomes. In references [11,94], the MV rule was employed to fuse multiple outputs (decisions) generated by basic classifiers.

This work introduces a fusion technique for various decision sources derived from a single hyperspectral image. The proposed approach leverages Markov random field and conditional random field graphical models due to their spatial regularization properties and their capacity to incorporate multiple decision sources in their energy functions. To achieve this, we suggest utilizing fractional abundances, taken through the sparse unmixing method SunSAL [95], as one of the decision sources. This is believed to offer an enhanced subpixel description in scenarios with mixed pixels and to be particularly suitable in situations with limited training data. While fractional abundances have been previously employed as features for direct hyperspectral image classification [96], or initially classified with a soft classifier that produces class probabilities for use in a decision fusion method [97], they have not been directly applied as a decision source within a decision fusion framework. Additionally, sparse representation classification (SRC) methods have been utilized.

Alongside the abundances, class probabilities from a probabilistic classifier (the MLR classifier) are generated. Initially, the MLR classifier takes reflectance spectra as input, but alternatively, contextual features can also be applied as input. Both decision sources (abundances and probabilities) offer two complementary perspectives on the hyperspectral image, providing a more comprehensive depiction of each pixel. This is expected to be advantageous, especially when dealing with limited training data. To amalgamate both decision sources, a decision fusion approach similar to the one proposed in [98] is adopted. To accomplish this goal, graphical models such as MRF or CRF are employed. These models incorporate spatial consistency constraints and cross-links between the two decision sources to guarantee coherence in their decisions. Additionally, the framework can be expanded to accommodate three or more decision sources.

### 3.1. MRF Regularization

In the conventional single-source MRF approach, a graph is constructed over a set of n observed pixels represented as $x=\left\{x_{1},\ldots ,x_{n}\;\right\}$ along with their corresponding class labels denoted as $y=\left\{y_{1},\ldots ,y_{n}\;\right\}$, which are associated with the nodes in the graph. The graph edges serve to capture the spatial neighborhood dependencies among the pixels. While the pixel values are already known, the task at hand is to estimate the labels. To achieve this, the primary objective is to maximize the joint probability distribution of the observed data and the labels, denoted as $P\left(x,y\right)$. In terms of energy-based formulations, the optimal labels are determined by minimizing the following energy function:

(24) $$E\left(y\right)=\sum_{i=1}^{n}\psi_{i}\left(y_{i}\right)+\beta \sum_{i=1}^{n}\sum_{j\in N_{i}}\psi_{i,j}\left(y_{i},y_{j}\right)$$

The unary potentials, denoted as $\psi_{i}\left(y_{i}\right)=-\ln\left(p\left(x_{i}\;|y_{i}\right)\right)$, are determined as the negative natural logarithm of the class conditional probabilities, which are represented as $p\left(x_{i}\;|y_{i}\right)$ [99]. In the context of high-dimensional data, an alternative formulation is employed: $\psi_{i}\left(y_{i}\right)=-\ln\left(\hat{p}\left(y_{i}\;|x_{i}\right)\right)$, where $\hat{p}\left(y_{i}\;|x_{i}\right)$ stands for the estimated posterior probabilities obtained through a probabilistic classifier [2,100]. As for the pairwise potentials, designated as $\psi_{i,j}$, they rely solely on label information and introduce smoothness constraints based on label similarity within the spatial neighborhood $N_{i}$ of pixel i. These pairwise potentials are defined as $\psi_{i,j}=\left(1-\delta \left(y_{i},y_{j}\right)\right)$, where $\delta \left(y_{i},y_{j}\right)$ represents the indicator function (δ(a, b)=1 for a=b and δ(a, b)=0 otherwise).

### 3.2. CRF Regularization

A limitation of the MRF method lies in its modeling of label neighborhood relationships independently of the observed data. In contrast, conditional random fields (CRFs) offer a set of advantageous characteristics that enhance flexibility and efficiency: 1. CRFs are discriminative models, directly estimating $P\left(y|x\right)$. 2. CRFs incorporate the observed data into their pairwise potential terms, enabling a more comprehensive consideration of the data when defining label relationships.

### 3.3. The Decision Sources

Let $x=\left\{x_{1},\ldots ,x_{n}\;\right\}$ represent a hyperspectral image comprising n pixels, where each $x_{i}\;\in \;R^{d}$ corresponds to the spectral bands. We have a training dataset, denoted as $D=\left\{\left(x_{1},y_{1}\right)\right\},\ldots ,\left(x_{m},y_{m}\right)\}$, containing m labeled samples with j=1,…, m, where each sample $x_{j}$ is associated with a label $y_{j}\in \;\left\{1,\ldots ,C\right\}$, where C represents the number of classes. The primary goal is to assign labels $y_{i}$ to each pixel $x_{i}$ in the image. Combining two sources of information is suggested for decision making. The first source uses the output probabilities generated by the multinomial logistic regression (MLR) classifier [101], which involves supervised classification based on spectral reflectance values. The second source of information comes from the sparse spectral simulation method known as SunSAL, as introduced in [95]. Regarding the initial source of information, the spectral values of the pixels are used as input to the MLR classifier. This enables us to find classification probabilities for each pixel $x_{i}$, which are represented as $p_{i}=p\left(x_{i}\right)=\left(p_{1}\left(x_{i}\right),\;\ldots ,p_{C}\left(x_{i}\right)\;\right)$, with

(25) $$p_{C}\left(x_{i}\right)=p\left(y_{i}=c|x_{i}\right)=\frac{exp\left(\beta_{c}^{T}x_{i}\right)}{\sum_{c=1}^{C}exp\left(\beta_{c}^{T}x_{i}\right)}$$

The regression coefficients $\beta_{c}\in R^{d}\;(c=1,\ldots ,\;C)$ are evaluated from the training data. To evaluate a class label from the probability vector, one can employ a maximum a posteriori (MAP) classifier, yielding ${\hat{y}}_{i}^{p}={argmax}_{c}p_{C}\left(x_{i}\right)$.

The second source of information involves computing the fractional abundances of each pixel $x_{i}$ using SunSAL. In this method, the training data serve as a dictionary of endmembers, denoted as $E=[x_{1},\ldots ,x_{m}]$ (in other words, the training pixels are assumed to represent pure materials):

(26) $$a^{*}=\left(a_{1}^{*},\ldots ,a_{m}^{*}\right)=arg{min}_{a}\frac{1}{2}{\left\|E_{a}-x_{i}\;\right\|}_{2}^{2}+\lambda {\left\|a\right\|}_{1},\;s.t.a\;\geq 0.$$

Subsequently, the obtained abundances that correspond to endmembers associated with class label $y_{j}=c$ are summed to yield a fractional abundance value denoted as $a_{c}\left(x_{i}\right)$ for each class c. This process contributes to the creation of an abundance vector, $a_{i}=a\left(x_{i}\right)=\left(a_{1}\left(x_{i}\right),\ldots ,a_{c}\left(x_{i}\right)\right)$. It is crucial to emphasize that these abundance values do not indicate the statistical probability of a pixel being accurately classified as part of class c. Instead, they signify the fractional presence of class c within the pixel. The incorporation of both decision sources in this manner results in a more comprehensive characterization of the pixels, which proves advantageous in scenarios involving data with many dimensions and limited training data.

Once the individual abundance values a and the probability outputs p have been obtained from the sparse unmixing and the MLR classifier, respectively, we proceed to perform decision fusion using MRF and CRF graphical models. These models employ compound energy functions that encompass inputs from the two available decision sources.

### 3.4. MRF Incorporating Cross-Links for Fusion (MRFL)

**Figure 3:**
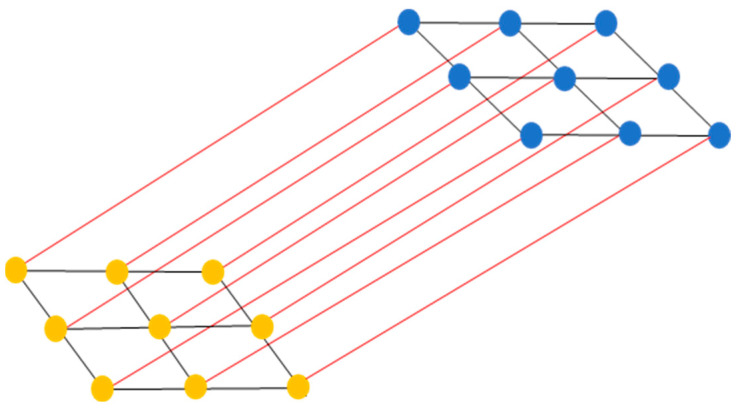
In the graphical interpretation of MRFL, the yellow nodes represent the random variables associated with $y^{a}$, while the blue nodes represent those associated with $y^{p}$. The black lines represent edges that capture spatial neighborhood dependencies, while the red lines represent cross-links between $y^{a}$ and $y^{p}$, encoding the potential interactions $\psi_{i,i}^{ap}\left(y_{i}^{a},y_{i}^{p}\right)$. The parameter γ controls the strength of influence of these interaction terms.

For each decision source, class labels are comparable. To enable the fusion of both decision sources, a bipartite graph is utilized that includes two types of nodes for each pixel. These nodes represent random variables linked to the labels $y_{i}^{a}$ and $y_{i}^{p}$, respectively. Within each type of node, edges are defined that capture the spatial dependencies among the pixels. Additionally, a cross-link linking both types of nodes is established; specifically, it links label $y_{i}^{a}$ with the corresponding label $y_{i}^{p}$ [102] (Figure 3).

The ultimate objective is to optimize the joint distribution encompassing the observed data and the associated labels from both sources: $P(a,\;p,\;y^{a},y^{p})$. In order to accomplish this requirement, the arising energy function is minimized:

(27) $$\begin{array}{l} E\left(y^{a},y^{p}\right)=\sum_{i=1}^{n}\psi_{i}^{a}\left(y_{i}^{a}\right)+\sum_{i=1}^{n}\psi_{i}^{p}\left(y_{i}^{p}\right)\\ \;\;\;\;\;\;+\beta [\sum_{i=1}^{n}\sum_{j\in \;N_{i}}\psi_{i,j}^{a}\left(y_{i}^{a},y_{j}^{a}\right)\\ \;\;\;\;\;\;+\sum_{i=1}^{n}\sum_{j\in \;N_{i}}\psi_{i,j}^{p}\left(y_{i}^{p},y_{j}^{p}\right)+\gamma \sum_{i=1}^{n}\psi_{i,i}^{ap}\left(y_{i}^{a},y_{i}^{p}\right)] \end{array}$$

The unary potentials are defined as follows: $\psi_{i}^{a}\left(y_{i}^{a}\right)=-\ln\left(a_{c}\left(x_{i}\right)\right)$ and $\psi_{i}^{p}\left(y_{i}^{p}\right)=-\ln\left(p_{c}\left(x_{i}\right)\right)$ for $y_{i}=c$. Here, $N_{i}$ represents a four-spatial neighborhood surrounding pixel i. Regarding the pairwise potentials from the individual sources, $\psi_{i,j}^{a}=\left(1-\delta \left(y_{i}^{a},y_{j}^{a}\right)\right)$ and $\psi_{i,j}^{p}=\left(1-\delta \left(y_{i}^{p},y_{j}^{p}\right)\right)$, these promote smoothness by considering label similarity within the spatial neighborhood of pixel i. These similarities in labels are derived from fractional abundances and classification probabilities, respectively. Additionally, the final pairwise term $\psi_{i,j}^{ap}=\left(1-\delta \left(y_{i}^{a},y_{j}^{p}\right)\right)$ penalizes disagreements between labels $y_{i}^{a}$ and $y_{i}^{p}$. Through these binary potentials, the Markov random field labeling (MRFL) model simultaneously accounts for spatial structuring and consistency between labels from the two decision sources. To solve this, the graph-cut a-expansion algorithm has been applied [91,103,104,105].

### 3.5. CRF with Cross-Links for Fusion (CRFL)

**Figure 4:**
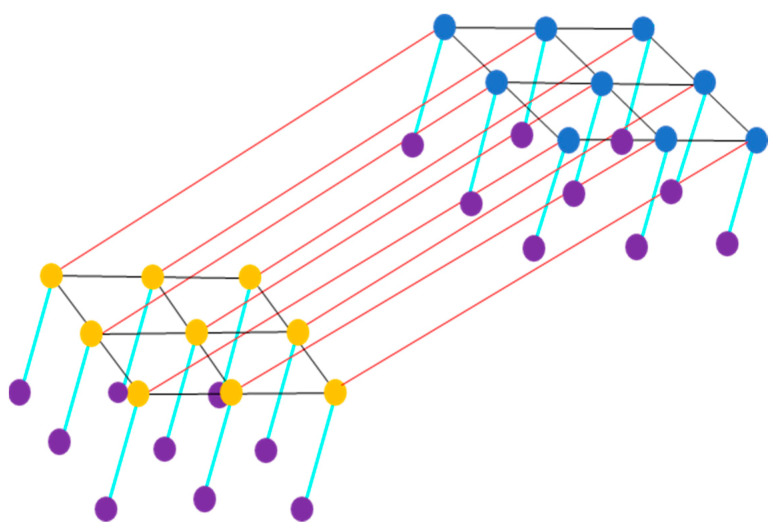
In the graphical illustration of CRFL, the purple nodes represent random variables linked to the observed data, the yellow nodes represent random variables related to the labels $y^{a}$ and the blue nodes represent random variables related to the labels $y^{p}$. The turquoise lines denote the connections between the labels and the observed data. The black lines represent edges that model spatial neighborhood dependencies, while the red lines represent cross-links between $\left(a,\;y^{a}\right)$ and $\left(p,\;y^{p}\right)$, encoding potential interactions $\psi_{i,i}^{ap}\left(\;y_{i}^{a},y_{i}^{p}|a,\;p\right)$. The parameter g controls the strength of influence of these interaction terms.

To make a difference from the previous approach, a discriminant method is used that extends the previous MRFL method. This alternative method directly models the posterior distribution $P\left(\;y^{a},y^{p}|a,\;p\right)$, simultaneously taking into account the correlations between the class labels $y^{a},y^{p}$ and the observed data a, p within the pair dynamics (see Figure 4).

The energy function is obtained as follows [98,102,106]:

(28) $$\begin{array}{l} E\left(y^{a},y^{p}|a,\;p\right)=\sum_{i=1}^{n}\psi_{i}^{a}\left(y_{i}^{a}\right)+\sum_{i=1}^{n}\psi_{i}^{p}\left(y_{i}^{p}\right)\\ \;\;\;\;\;\;+\beta \left[\sum_{i=1}^{n}\sum_{j\in \;N_{i}}\psi_{i,j}^{a}\left(y_{i}^{a},y_{j}^{a}|a_{i,}a_{j}\right)+\sum_{i=1}^{n}\sum_{j\in \;N_{i}}\psi_{i,j}^{p}\left(y_{i}^{p},y_{j}^{p}|p_{i,}p_{j}\right)\right]\\ \;\;\;\;\;\;+\gamma \sum_{i=1}^{n}\psi_{i,i}^{ap}\left(y_{i}^{p},y_{i}^{p}|a_{i,}p_{i}\right) \end{array}$$

The unary terms are similar to those in the MRFL model. A contrast-sensitive Potts model is employed for the pairwise potentials:

(29) $$\psi_{i,j}^{a}\left(y_{i}^{a},y_{j}^{a}|a_{i,}a_{j}\right)=\left(1-\delta \left(y_{i}^{a},y_{j}^{a}\right)\right)exp(-\frac{{\left\|a_{i-}a_{j}\right\|}_{2}^{2}}{\sigma^{a}}),$$

(30) $$\psi_{i,j}^{a}\left(y_{i}^{a},y_{j}^{a}|p_{i,}p_{j}\right)=\left(1-\delta \left(y_{i}^{a},y_{j}^{a}\right)\right)exp(-\frac{{\left\|p_{i-}p_{j}\right\|}_{2}^{2}}{\sigma^{p}}),$$

(31) $$\psi_{i,j}^{a}\left(y_{i}^{a},y_{i}^{a}|a_{i,}p_{i}\right)=\left(1-\delta \left(y_{i}^{a},y_{i}^{a}\right)\right)exp(-\frac{{\left\|a_{i-}a_{j}\right\|}_{2}^{2}}{\sigma^{ap}}).$$

The initial component of the energy function incentivizes adjacent pixels with comparable abundance vectors to be assigned to the same class. The subsequent component advocates for allocating neighboring pixels with akin class probabilities to the same class. Lastly, the third component encourages the assignment of similar class labels, $y_{i}^{a}$ and $y_{i}^{p}$, to pixels when their abundance vector closely resembles the probability vector. The parameters s are standard deviations that govern the strength of these influences. To optimize this energy function, the graph-cut a-expansion algorithm is employed. Proposed methodologies utilize the graph-cut a-expansion algorithm [91,103,104,105], which exhibits a worst-case computational complexity of $O({mn}^{2}\left|P\right|)$ for a single optimization problem. Here, m represents the number of edges, n denotes the number of nodes in the graph and |P| signifies the cost of the minimum cut. Therefore, the theoretical computational complexity of the proposed method is expressed as $O(kC{mn}^{2}\left|P\right|)$, with k being the maximum number of iterations and C signifying the number of classes.

In summary, two innovative decision fusion methodologies are introduced for hyperspectral image classification in the context of remote sensing. These methods address the challenges posed by high dimensionality, limited ground truth information, mixed pixel content and spectral collinearity in real-world scenarios. The decision fusion framework relies on probabilistic graphical models, specifically MRFs and CRFs, and leverages a combination of complementary decision sources: 1. Fractional abundances, derived through sparse unmixed pixels, enhance the characterization of subpixel content, especially in mixed pixels. 2. Probabilistic outputs from a soft classifier provide confidence levels regarding the spectral content of the pixels. These approaches interpret two fundamental types of relationships among the underlying variables: (a) spatial dependencies among pixels and (b) consistency between the decisions made by two distinct information sources. This dual consideration enables a more comprehensive analysis, incorporating both the spatial context of pixels and the agreement between different decision sources. Fractional abundances have proven to be informative decision sources, and both MRFL and CRFL methods outperform additional fusion approaches when used in the same decision sources. CRFL demonstrates high overall accuracy and robustness across a wide range of parameter values. Furthermore, the inclusion of a third decision source enhances classification accuracies.

## 4. Integrating MODIS and Landsat Data for Land Cover Classification by Multilevel Decision Rule

Land cover (LC) mapping plays a crucial role in environmental planning and management by monitoring changes in land cover over time. With the rapid advancement of remote sensing technology in recent decades, various classification methods have been developed to create accurate LC maps [107] using a wide range of remote sensing data types, including multi-resolution optical data and SAR data. However, it is important to note that there is no one-size-fits-all solution, as neither a single classification method nor a specific type of data is universally optimal for all scenarios [72].

To enhance the accuracy of LC mapping, data fusion has emerged as a promising approach to leverage the complementary strengths of multiple data sources. In the literature, data fusion is categorized into three levels: the pixel level, the feature level and the decision level (or symbol level) [108]. Pixel-level fusion involves merging measured physical parameters obtained from remote sensors. Feature-level fusion, on the other hand, begins by extracting features, like texture or spectral information, from images and then merges these features from sources for which the confidence is higher. The decision-level fusion approach, which is based on symbols, is commonly used in classifier combination. It represents the utmost degree of data fusion and involves combining preliminary classified results from individual classifiers or classified data [29]. Decision fusion employs various fusion strategies, such as MV [1], weighted average (WA) [109], Bayesian reasoning (BR) [110] and Dempster–Shafer evidence theory (DS) [111].

MODIS and Landsat data [48] are widely employed in LC classification due to their fine temporal and spatial resolutions, as well as their availability at no cost. The combination of data with high spatial and temporal resolution is particularly advantageous for improving LC classification accuracy, especially in regions prone to cloud cover and rainfall, which often obscures high-spatial-resolution data, making it challenging to extract continuous surface information [112]. Surprisingly, there has been limited research focused on combining MODIS and Landsat LC information through decision fusion.

In general, the process of merging data from multiple sensors through decision fusion involves two main steps. Initially, the images from each sensor are classified individually using certain classifiers. Second, the outputs of these classifiers are integrated using a different combination function.

Considering the flexibility of decision-level fusion, it is possible to fuse data from these two satellites at the decision level. However, due to the mixed nature of information in MODIS data, a specialized fusion scheme is required to combine MODIS and Landsat data effectively. In this paper, an innovative multilayer decision fusion scheme is designed to merge MODIS and Landsat dataset information. This model consists of three tiers: the Landsat pixel layer at 30 m, the object layer, and the MODIS pixel layer at 250 m. The object layer is created by performing multi-resolution segmentation of Landsat pixels, with segmentation confined within MODIS pixels. Each layer offers a membership degree for each considered class. To combine these layers, a weighted measure is utilized that accounts for both local and global confidence mechanisms. The fundamental class decision method adopts the compromise combination approach introduced by Fauvel et al. [72]. This decision fusion occurs across three layers, involving the MODIS pixel–object layer and the object–Landsat pixel layer. The ultimate result is an enhanced classification accuracy when compared to the straightforward combination of coarse-to-fine-resolution data.

### 4.1. Comprehensive Fusion Strategy

**Figure 5:**
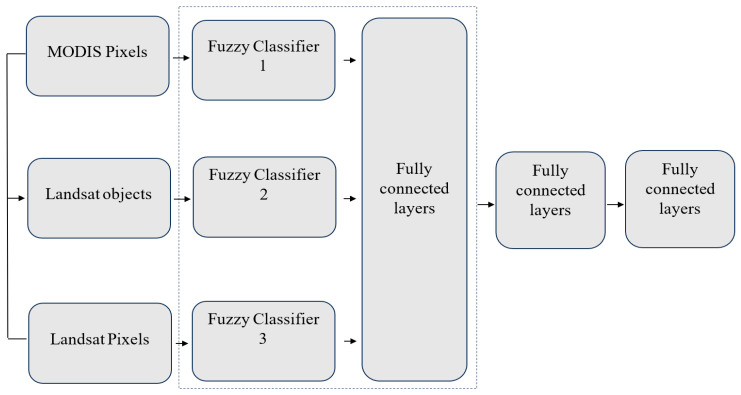
The comprehensive fusion strategy employed in the methodology.

The overall process of multilevel decision fusion is depicted in Figure 5 and can be delineated into two distinct phases: the fuzzy classification phase and the decision fusion phase. In the first phase, fuzzy classification was applied to the MODIS data by means of an approach that employs time series measures of similarity, while fuzzy classification for Landsat data was accomplished using a nearest neighbor classifier. Additionally, Landsat data were subjected to object-oriented classification. In the second phase, once the memberships of the three-level data were obtained, confidence assessments were conducted both locally and globally. Subsequently, the memberships derived from the three-tiered data, along with assessments of local and global confidence, were integrated by properly fusing decisions. Following this fusion process, the fuzzy classification outcomes of the three-tiered data were amalgamated, considering the efficacy of an individual classifier.

### 4.2. Fuzzy Classification and Operation

Fuzzy Aggregation Operators

When dealing with uncertain or fuzzy objects or classes, the concept of fuzziness can be incorporated into the classification process. In this context, a fuzzy set F within a reference set U is defined by a membership function $\mu_{F}$, where $\mu_{F}:U\to [0,1]$. Here, $\mu_{F}=0$ indicates that μ unequivocally does not belong to fuzzy set F, while values between 0 and 1, such as $0<\mu_{F}<1$, indicate that μ is partially associated with F. Consider two fuzzy sets, F and G, within the set U, each characterized by membership functions $\mu_{F}$ and $\mu_{G}$ [113]. Fusion operators, which encompass decision operators, combination operators and cut operators, are rooted in classical fuzzy set operations. Taking into account conflicts stemming from diverse information sources, the compromise combination operation is delineated as follows:

(32) $$C\left(\mu_{F}\left(x\right),\mu_{G}\left(x\right)\right)={sup}_{x}min\left(\mu_{F}\left(x\right),\mu_{G}\left(x\right)\right)$$

Several other flexible combination operators that have been customized to this context have been suggested, including the prioritized fusion operator [72,114]:

(33) $$\mu \left(x\right)=min\left(\mu_{F}\left(x\right),\;max\left(\mu_{G}\left(x\right),1-C\left(\mu_{F}\left(x\right),\mu_{G}\left(x\right)\right)\right)\right)$$

(34) $$\mu \left(x\right)=max\left(\mu_{F}\left(x\right),\;min\left(\mu_{G}\left(x\right),C\left(\mu_{F}\left(x\right),\mu_{G}\left(x\right)\right)\right)\right)$$

Nearest Neighbor Classification

Among supervised classification methods, the NN classifier stands out as the most frequently employed fuzzy classification technique. NN classification relies on the concept of minimum distance within a nearest neighbor feature space, where training data are assembled using spectral, shape, or texture feature values. The determination of distance within this NN feature space is accomplished through a straightforward Euclidean distance (ED) function:

(35) $$d\left(x,y\right)={\left(\sum_{i=1}^{m}{\left(x_{i}-y_{i}\right)}^{2}\right)}^{1/2}$$

where the distance metric d(x, y) represents the Euclidean distance (ED) between the samples to be classified. Smaller values of ED indicate a higher degree of similarity between the data and the reference samples. It is worth noting that these Euclidean distances offer the opportunity to convert the features into fuzzy membership numbers, which fluctuate in the range of 0 to 1.

Classification of Image Objects

The middle level of the fusion scheme serves the purpose of bridging the gap between MODIS pixels and Landsat pixels through segmented objects in the image. The incorporation of an object-level decision is advantageous because object features encompass a richer set of information, including neighborhood and texture details, which are valuable for the fuzzy classification process. Furthermore, the object level comprises a stack of homogeneous pixels, making it a more logical choice for fusion with MODIS pixels. The image segmentation is carried out using multi-resolution segmentation (MRS) within the e-Cognition platform. It is important to note that the segmentation is specifically restricted to MODIS pixels, as depicted in Figure 5. Within the e-Cognition platform, MRS relies on five parameters to control the segmentation outcome: scale, shape, color, compactness and smoothness. Among these parameters, the scale parameter, which governs the size of resulting polygons, holds paramount importance. Optimal segmentation involves finding an equilibrium among polygon size, internal consistency within an object and dissimilarity between objects. The allocation of relative weight to shape and color criteria during segmentation is governed by the shape and color parameters. A higher shape value reduces the influence of color on segmentation. Regarding compactness and smoothness criteria, higher weight values lead to the formation of more compact image objects. Following the segmentation process, the objects undergo classification using sample points. The fuzzy classification procedure is also executed within the e-Cognition software (Version 9.4) Object-related information, encompassing spectral information, texture, shape and distinctions from neighboring objects, is input into an NN feature space for sample training.

Time Series Similarity

Temporal trajectory analysis is a valuable approach for extracting meaningful patterns from multi-temporal sequences, with time series similarity serving as a crucial metric in this context [115]. Vegetation exhibits a seasonal temporal trajectory influenced by plant phenology [116]. The Vegetation Index (VI), extracted from satellite data, plays a critical role in monitoring and assessing the conditions of vegetation growth. Additionally, VI has proven effective in distinguishing various land cover (LC) types [117]. Consequently, employing VI time series similarity measurements proves to be a robust method for land cover classification. In accordance with the linear spectral unmixing theory, the VI time series often reflects dominant LC types. In cases where the landscape is heterogeneous, the VI time series tends to resemble the average VI time series associated with the LC types.

The initial step involves constructing the MODIS normalized difference vegetation index (NDVI) time series. Assuming there are N pixels within an image and M layers of MODIS NDVI imagery obtained throughout a year in chronological order, commencing with the first day of the year, each pixel possesses two attributes: its coordinates (x, y) and an NDVI sequence defined as follows: $Sm=\left\{\left(\left(x_{q},y_{q}\right),\;{VI}_{q}^{l}\right),q=1,\ldots ,N;l=1,\ldots ,M\right\}$, where $\left(x_{q},y_{q}\right)$ represents the coordinates of each pixel and ${VI}_{q}^{l}$ corresponds to the NDVI values in each layer of the MODIS time series data. Subsequently, the reference time series for each land cover type is selected. Due to variations in reflectance, different LC types exhibit distinct shapes in their VI time series. The standard VI time series typically come from the pixel values in satellite images or ground truth information. The next step involves calculating the similarity between a pixel’s VI time series and the reference VI time series, based on the Euclidean distance (ED) principle. ED is defined as the cumulative distance between corresponding pointwise values on two curves (Equation (36)):

(36) $$ED=\sum_{l=1}^{M}abs\left({VI}_{1}^{l}-{VI}_{2}^{l}\right)$$

Here, ED represents the Euclidean distance between the curves ${VI}_{1}^{l}$ and ${VI}_{2}^{l}$, with M representing the number of points on these curves. The final step involves obtaining normalized memberships based on the calculated ED value. Smaller ED values indicate a higher degree of similarity between the two curves. Consequently, the memberships for MODIS data are determined using Formula (1)—normalized Euclidean distance (NED). Equation (37) outlines the computation of NED.

(37) $$NED=(ED-{min}_{ED})/({max}_{ED}-{min}_{ED})$$

### 4.3. Uncertainty and Decision

Pointwise Global

The fundamental class decision approach utilized in this context is based on the compromise combination method introduced by Fauvel and Benediktsson [72]. It operates under the premise that a membership is deemed “trustworthy” when it displays minimal fuzziness. Essentially, a dependable fuzzy set should possess a membership considerably higher than others. Conversely, if membership values within a set are tightly grouped, the classifier is characterized as “untrustworthy”. The quantification of fuzziness is defined as follows:

(38) $$H_{aQE}\left(\mu_{F}\right)=\frac{1}{{n2}^{-2a}}\sum_{i=1}^{n}{\mu_{F}\left(x_{i}\right)}^{a}{\left(1-\mu_{F}\left(x_{i}\right)\right)}^{a}$$

where the value of a is 0.5 [40]. Subsequently, to assign how the weights influence the different fuzzy sets, each fuzzy set is weighted using the following formula:

(39) $$\left\{\begin{array}{c} \omega_{i}=\frac{\sum_{k=0,k\neq i}^{m}H_{aQE}\left(\mu_{F}\right)}{\left(m-1\right)\sum_{k=0}^{m}H_{aQE}\left(\mu_{F}\right)}\\ \sum_{i=1}^{m}\omega_{i}=1 \end{array}\right.$$

where $E_{v}(\mu_{k}(\mu_{i}))$ describes the degree of fuzziness for source k, where m represents the number of sources. The value of $\omega_{i}$ tends to approach 1 when a source exhibits a low level of fuzziness.

Overall Accuracy

The uncertainty of membership of the local context can be described as one aspect of the measurement uncertainty. Overall accuracy means the accuracy of classification by every classifier on the entire image. In Equation (40), notice the class-wise measure of accuracy $\left(CA_{i}\right)$:

(40) $$CA_{i}=\frac{2*{pr}_{i}*{tp}_{i}}{{pr}_{i}+{tp}_{i}}$$

where ${tp}_{i}$ represents the true positive rate (TPR), indicating the percentage of samples correctly classified into class i among all samples that truly belong to class i. Additionally, ${pr}_{i}$ signifies precision, denoting the percentage of samples that genuinely pertain to class i among all samples classified as class i. Considering the interconnectedness of MODIS data classification accuracy with the area proportion, it is essential to modify the global accuracy of MODIS data by incorporating an area factor $\left(A^{p}|p=1,\;2,\;3,\ldots ,\;10\right)$. This factor accounts for the accuracy of graded area proportions, ranging from 10% to 100%. As a result, Equation (40) is modified as follows:

(41) $${CA}_{i}^{p}=\left(A_{i}^{p}*CA_{i}*10\right)/\sum_{p=1}^{10}A_{i}^{p}$$

Decision Rule

Decision fusion is achieved by adapting the local environment using a context-dependent compromise method. Research has shown that this fusion approach prioritizes the most reliable source by adjusting for local context:

(42) $$\mu_{f}^{j}\left(x\right)=max\left(min\left(\omega_{i}\mu_{i}^{j}\left(x\right),f_{i}^{j}\left(x\right)\right),i\in \left[1,m\right]\right)$$

where $f_{i}^{j}\left(x\right)$ represents the global confidence of source (classifier) i for class j; $\omega_{i}$ is the local context defined in Equation (37); $\mu_{i}^{j}$ is an element of the membership, indicating a membership value assigned to class j; and m is set to 2. For the calculation of $f_{i}^{j}\left(x\right)$, the average class-wise accuracy introduced in the previous paragraph is utilized ($CA_{i}$ and ${CA}_{i}^{p}$) and. To derive the global classification accuracy of MODIS data, denoted as $f_{m}^{j}\left(x\right)$, Equation (41) is employed to compute the pointwise accuracy of the classified data. Similarly, the object layer’s global classification accuracy, $f_{o}^{j}\left(x\right)$, and the Landsat pixel layer’s global classification accuracy, $f_{l}^{j}\left(x\right)$, are determined using Equation (40) and sampling points. During the fusion phase, the local confidence is initially determined using Equations (38) and (39). These local confidence values for MODIS membership, object membership and Landsat membership are represented as $\omega_{m}$, $\omega_{o}$ and $\omega_{l}$, respectively. The last fusion method is presented in Equation (43):

(43) $$\mu_{mol}^{j}\left(x\right)=max\left(min\left(\omega_{m}\mu_{m}^{j}\left(x\right),f_{m}^{j}\left(x\right)\right),min\left(\omega_{o}\mu_{o}^{j}\left(x\right),f_{o}^{j}\left(x\right)\right),min\left(\omega_{l}\mu_{l}^{j}\left(x\right),f_{l}^{j}\left(x\right)\right)\right)$$

Finally, an improvement of about 7% occurred in the overall accuracy of the test. Additionally, it is certain that the decision fusion of three layers is more accurate than the MODIS–object decision with two layers.

## 5. Decision Fusion of Optical and SAR Images [77]

During the past few decades, there has been a growing focus on monitoring climate change. This study aims to develop high-resolution (10–30 m) land cover mapping products for three subcontinental regions of climate significance [118]. However, the combination of multispectral and multitemporal data, high spatial resolution and large geographical coverage presents significant computational challenges. When it comes to discriminating between different land covers, optical and SAR sensors are known to exhibit complementary behaviors [119]. Optical imagery is conventionally the primary data source for LC mapping, while SAR data can provide valuable insights into specific land cover types like urban areas and water bodies, despite being affected by speckle noise. Given the LC maps generated separately using optical and SAR data, decision fusion is employed to combine the probabilistic decisions from these sources into a final result by giving importance to the level of uncertainty associated with each source.

This research proposes a Bayesian decision fusion approach for multi-sensor optical–SAR image classification, coupled with an MRF model to account for the spatial–contextual information inherent in the high-resolution input data. The primary focus is on LC mapping at high spatial resolution, specifically 10 m, using Copernicus Sentinel imagery, while striving to keep the computational burden low to facilitate application over large subcontinental regions. Posterior probabilities obtained through generative classifiers applied to optical and SAR data are fused to generate the final classification map. Notably, the key contributions of this approach are twofold. Firstly, it introduces a specific Bayesian fusion rule to handle cases where the sets of classes used by the two individual classifiers do not match. This aligns with the varying importance of optical and SAR data in distinguishing different classes, a crucial consideration in large-scale applications characterized by diverse LCs. Secondly, the study presents a case-specific sequential formulation of the iterated conditional mode (ICM) algorithm for MRF energy minimization. This tailored ICM formulation is based on convolution operators and is designed to reduce computational demands within a conventional Python-based environment.

### 5.1. Fusion with Partially Overlapping Sets of Classes

Consensus theory, as outlined in [120,121], encompasses general procedures aimed at combining multiple probability distributions to synthesize their estimates. The fundamental challenge lies in consolidating different viewpoints, symbolized by the fusion of posterior probabilities from various classifiers, each associated with a specific data source. If all the classifiers generate Bayesian outputs, and therefore their predictions are characterized probabilistically, the primary objective is to generate a single probability distribution that encapsulates their collective estimates.

Consider a scenario where optical and SAR images are captured over the same geographical area. Let O and S represent the optical and SAR feature vectors for a given pixel, respectively. Let us assume that two distinct generative models have independently computed posterior probabilities based on either O or S. Define $\Omega_{C}$ as the set of common classes considered by both classifiers, and $\Omega_{O}$ and $\Omega_{S}$ as the sets of classes exclusively distinguished by the classifiers operating on O and S, respectively. Consequently, the optical and SAR classifiers work on $\Omega_{O}\;\cup \;\Omega_{C}$ and $\Omega_{S}\;\cup \;\Omega_{C}$, respectively, while the overall set of classes encompasses $\Omega =\Omega_{O}\;\cup \;\Omega_{C}\;\cup \;\Omega_{S}$. Let x=[O,S] denote the complete data vector for a typical pixel, and let $\omega_{j}$ represent the j-th information class $\left(\omega_{j}\;\in \;\Omega \right)$. A well-established and often effective consensus rule is the LOGP [120,121]:

(44) $$L(\omega_{j}\;|x,\Omega_{C})=a_{j}lnP(\omega_{j}\;|O,\Omega_{C})+\beta_{j}lnP(\omega_{j}\;|S,\Omega_{C})$$

We have two generative models that estimate pixelwise posteriors denoted as $P(\omega_{j}\;|O,\Omega_{C})$ and $P(\omega_{j}\;|S,\Omega_{C})$. These models offer insights into the probability distribution for each class $\omega_{j}$. Additionally, we have parameters $a_{j}$ and $\beta_{j}$, which represent per-class weights that reflect the discriminatory capabilities of each sensor towards $\omega_{j}$. While the function L(·) produces a result through probabilistic fusion, it does not inherently yield values within the [0, 1] interval. To address this and obtain a probabilistic output that can be interpreted as a fused posterior probability $P_{F}(\omega_{j}\;|x,\Omega_{C})$, a softmax operator is employed:

(45) $$P_{F}\left(\omega_{j}\;|x,\Omega_{C}\right)=\frac{expL(\omega_{j}\;|x,\Omega_{C})}{\sum_{\omega_{k}\in \Omega_{C}}L(\omega_{k}\;|x,\Omega_{C})}$$

The probability function $P_{F}\;(\cdot )$ is originally conditioned to the subset of classes $\Omega_{C}$. To generalize it to the entire set of classes, the full posterior probability, which is not conditioned to $\Omega_{C}$, is computed by applying the total probability theorem:

(46) $$\begin{array}{l} P_{F}(\omega_{j}|x)=P(\omega_{j}|x,\Omega_{C})P(\Omega_{C}|x)+P(\omega_{j}|x,\Omega_{O})P(\Omega_{O}|x)\\ \;\;\;\;\;+P(\omega_{j}|x,\Omega_{S})P(\Omega_{S}|x)\\ \;\;\;\;\;=PF\;(\omega_{j}|x,\Omega_{C})P(\Omega_{C}|x)+P(\omega_{j}|O,\Omega_{O})P(\Omega_{O}|x)\\ \;\;\;\;\;+P(\omega_{j}|S,\Omega_{S})P(\Omega_{S}|x), \end{array}$$

where the following conditional independence assumptions are made: $P(\omega_{j}\;|x,\Omega_{S})=P(\omega_{j}|S,\Omega_{S});\;P(\omega_{j}|x,\Omega_{O})=P(\omega_{j}|O,\Omega_{O})$. These assumptions align with the notion that the classes within $\Omega_{O}$ (and similarly within $\Omega_{S}$) are solely distinguished through the analysis of O (respectively, S). In the presented methodology, the combined posterior probabilities for the three distinct sets of thematic classes, namely $\Omega_{O}$, $\Omega_{S}$ and $\Omega_{C}$, are modeled in the following manner:

(47) $$\begin{array}{l} P(\Omega_{O}|x)=\lambda P(\Omega_{O}|O,\Omega_{O}\cup \Omega_{C}),\;P(\Omega_{S}|x)\\ \;\;\;\;\;\;=(1-\lambda )P(\Omega_{S}|S,\Omega_{S}\;\cup \;\Omega_{C}),\;P(\Omega_{C}|x)\\ \;\;\;\;\;\;=\lambda P(\Omega_{C}|O,\Omega_{O}\;\cup \;\Omega_{C})+(1-\lambda )P(\Omega_{C}|S,\Omega_{S}\;\cup \Omega_{C}) \end{array}$$

The chosen parameter λ, which satisfies 0 ≤ λ ≤ 1, ensures that the resulting terms sum up correctly to unity (∀λ∈ [0, 1]). This choice allows for the effective combination of probabilistic outputs from both optical and SAR sensors using a linear opinion pool approach, particularly for the shared classes. Moreover, it allows the representation of exclusive classes as functions of the output produced by one of the two single-sensor classifiers. In cases where $\Omega_{S}=\emptyset$, a desirable choice is λ=1, and conversely, when $\Omega_{O}=\emptyset$, λ should be set to 0. To strike a balance and cover both limit cases, a suitable weight can be calculated as $\lambda =P(\Omega_{O})/[P(\Omega_{O})+P(\Omega_{S})]$, with the prior probabilities $P(\Omega_{O})$ and $P(\Omega_{S})$ being estimated from the training set.

### 5.2. Fast Formulation of ICM

Consider a scenario where I represents the pixel lattice and $y_{i}$ represents the class label assigned to the i-th pixel (where $\left(y_{i}\;\in \;\Omega ,\;i\;\in \;I\right)$). To facilitate local contextual information, a neighborhood system is established, ${\left\{\partial_{i}\right\}}_{i\in I}$, which associates each i-th pixel with a set $\partial_{i}\;\subset \;I$, consisting of neighboring pixels [122]. In this context, $\partial_{i}$ corresponds to the first-order (four-connected) neighborhood [122]. For modeling the local contextual information, a Potts MRF model is adopted. This model is characterized by the following local posterior energy function:

(48) $$U\left(y_{i}|x_{i},y_{\partial_{i}}\right)=-\log P_{F}\left(y_{i}|x_{i}\right)-\gamma \sum_{j\in \partial_{i}}\delta \left(y_{i},y_{j}\right)$$

Here, $y_{\partial_{i}}$ represents $\left\{y_{i}\right\}$ for $j\in \partial_{i}$, γ is a positive weight and δ(·) is the Kronecker delta.

In the context of applying MRF-based techniques to large images, the crucial task is to minimize the energy function U concerning the random field Y, which represents the class labels. This is especially important given the substantial computational time required to process extensive datasets. In this regard, the ICM algorithm strikes an efficient balance between accuracy and computational workload [123]. ICM operates by iteratively updating the label of each pixel as $y_{i}\;\leftarrow \;{argmin}_{\omega_{j}\in \Omega }\;U(\omega_{j}|x_{i},y_{\partial_{i}})$. The advantage of ICM lies in its ability to ensure quick execution times. However, the conventional formulation of ICM often comes with limitations, particularly in terms of computational efficiency, as it necessitates a scan of the entire image to evaluate the energy for each class for each pixel separately. This scanning process can be time-consuming. To address this issue, a specialized ICM formulation is proposed to reduce execution time, albeit at the expense of slightly higher memory requirements.

**Figure 6:**
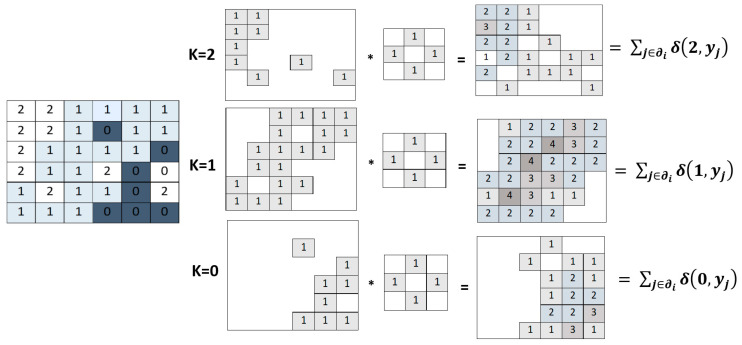
Convolution procedure applied to the obtained binary images in order to help with voting.

The proposed approach involves a reformulation of the minimum energy problem, making it feasible to utilize convolutions for evaluating the requisite energy terms. A convolution mask is defined to mirror the structure of the pixel neighborhood. In this mask, a value of 1 is assigned to the elements corresponding to the central element’s neighbors, while all other elements are set to zero. The application of convolution in the context of the Potts MRF is designed to conduct a vote count within the neighborhood. This count represents how many neighboring pixels advocate for a specific class assignment to the central pixel. To formalize this concept, the label image is divided into a series of binary images, each corresponding to one of the K=|Ω| classes. In the k-t map, the i-th pixel is assigned a value of 1 if $y_{i}=k\;(k=1,\;2,\ldots ,\;K)$. While Figure 6 illustrates a simple example with k=3, the same principle applies to any number of classes. By applying convolution between the neighboring mask and the pre-defined binary images, we can determine how many neighboring pixels endorse each potential class change. Consequently, all the necessary data to execute a single iteration of ICM (48) can be obtained through a single convolution operation on the stack of binary images.

In conclusion, when addressing the classification of large images, computation time is of paramount importance. A large-scale optical–SAR decision fusion method has been introduced, based on consensus theory and Markov random fields. This method encompasses both classes shared among individual decision sources and single-source classes. It also incorporates a specific ICM formulation that prioritizes efficient computation when dealing with extensive imagery.

## 6. SAR Image Fusion Classification Based on the Decision-Level Combination of Multi-Band Information [71]

Single-band SAR images provide limited target information, while multi-band SAR systems offer the ability to perform high-resolution imaging across multiple bands simultaneously [124,125]. This multi-band approach allows for a more comprehensive description of surface characteristics. By merging the classification outcomes from multi-band SAR images, we can achieve a more accurate and dependable classification outcome compared to using single-band image data alone. In recent decades, SAR image classification has seen significant advancement [126,127,128,129,130]. Existing algorithms for SAR image classification can be broadly categorized into three groups based on whether labeled data are utilized in the learning approaches for training: unsupervised, semi-supervised and supervised.

In this paper, to effectively leverage the complementary characteristics of multi-band classification information for the SAR image classification of a given scene, an innovative decision fusion technique, called the SAR image classification method, based on the decision-level combination of multi-band information is introduced. In this suggested approach, the DS theory [131,132,133] is used to model the uncertainty associated with the classification outcome of each pixel and to merge the classification results from multiple-band SAR images. In the beginning, multi-band SAR image data are gathered from sensors and then input into a CNN to obtain single-band classification results. Subsequently, the belief entropy [134] is computed for the classification of each pixel to assess the uncertainty associated with the classification. A basic probability assignment (BPA) is generated for each band after normalization. Then, leveraging the concepts of term frequency–inverse document frequency (TF-IDF) [135,136] and neighborhood influence, the overall weight is calculated for each band of every pixel to realize a combination of mean weights of BPAs from various band images. Finally, the classification outcome is derived based on the combined BPA. This approach utilizes decision fusion within the framework of evidence theory to quantify the uncertainty of classification results across different bands. The evidence combination technique is employed to integrate classification results from various bands, thereby reducing uncertainty and enhancing classification accuracy. A key challenge in the decision fusion process is assessing the complementarity between items of evidence. To address this, the notion of TF-IDF text mining is introduced into the conflict coefficient. This novel method for measuring the similarity of evidence, combined with neighborhood information, effectively quantifies complementarity between pixels, resulting in more precise decision fusion outcomes.

### 6.1. Single-Band SAR Image Classification Based on CNN

The CNN utilizes a network structure with weight sharing to efficiently decrease the quantity of weights and connections across various layers. Within the convolutional layers of the CNN, the primary task is to perform convolution operations, allowing for the extraction of image features through these operations. The convolution operation entails sliding convolution kernels across the input matrix to calculate the dot product within the current region. Repeatedly performing this process yields convolutional results.

Following the convolutional layers, pooling layers are connected to reduce the size of the extracted features, emphasizing the most pertinent information. Maximum pooling selects the maximum value within the current scanning area, while average pooling computes the average value of the current scanning area.

To integrate the features extracted from the preceding layer and facilitate classification, a fully connected layer is employed. The number of outputs in this layer matches the classification category count, with all nodes in the fully connected (FC) layer being connected to the previous layer.

**Figure 7:**
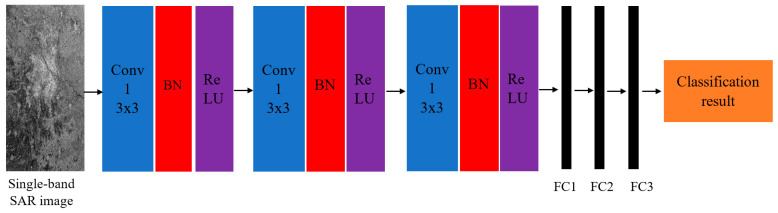
The architecture for a single-band SAR classification network.

The CNN structure designed for single-band SAR image classification is illustrated in Figure 7, comprising three convolutional modules and three FC layers.

A convolution kernel of 3×3, a BatchNorm layer and a rectified linear activation function (ReLU) layer comprise each convolutional module contained in a convolutional layer. Its output is

(49) $${Out}_{1}=ReLU(BN(\;f_{1}(x)))$$

(50) $${Out}_{2}=ReLU(BN(\;f_{2}({Out}_{1})))$$

(51) $${Out}_{3}=ReLU(BN(\;f_{3}({Out}_{2})))$$

where x represents the input and ${Out}_{k}$ means the output of the k-th convolutional unit, where k=1, 2, 3. $f_{k}$ denotes the convolutional function, “BN” refers to the function BatchNorm and ReLU means activation mode.

For the classification, entirely connected layers are used. The output of three FC layers is

(52) $$Out={FC}_{3}({FC}_{2}({FC}_{1}({Out}_{3})))$$

where ${FC}_{k}$ is the FC layer, k=1, 2, 3.

Patches of the single-band SAR image are partitioned and input into the network. Subsequently, the network generates pixel-level classification outcomes from the ultimate FC layer.

### 6.2. Method for SAR Image Classification through Decision-Level Fusion of Multi-Band Information

**Figure 8:**
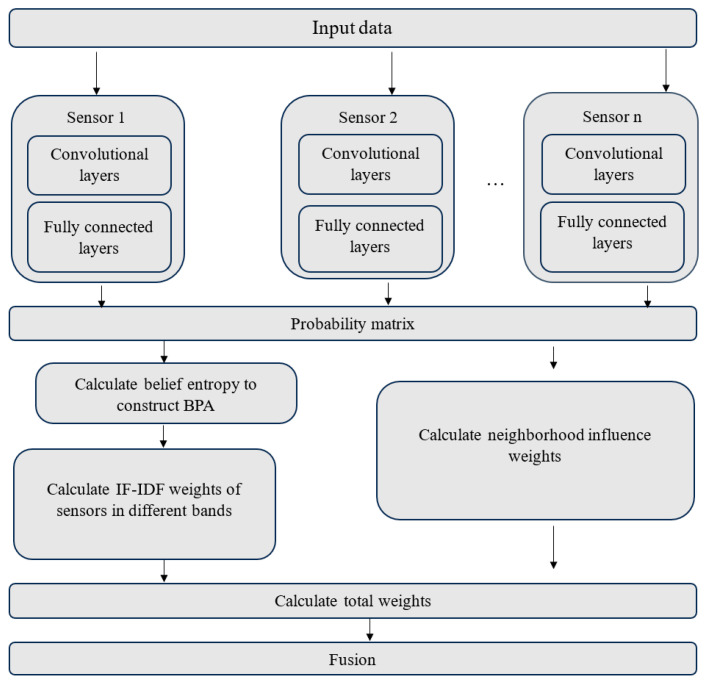
The flowchart of the SAR image classification method that relies on the decision-level integration of multi-band information.

Suppose we have a variety of sensors operating in different wavebands, which can be denoted as $X=\left\{x_{1},x_{2},\ldots ,x_{n}\right\}$. After the images acquired from these sensors undergo classification, we end up with h categories for each pixel $u_{ij}$, represented as $\Theta =\left\{\theta_{1},\theta_{2},\ldots ,\theta_{h}\right\}$. A flowchart illustrating the SAR image classification method that relies on the decision-level fusion of multi-band information is presented in Figure 8.

As depicted in Figure 8, the classification outcomes for each pixel within a single-band SAR image are represented as a probability matrix. To assess the reliability of various pieces of evidence, Shannon entropy is employed for the belief entropy. It calculates the belief entropy for each pixel’s classification within the probability matrix to gauge the classification’s uncertainty, resulting in a basic probability assignment (BPA) for each band. Taking inspiration from the TF-IDF concept, weights for various sensors are calculated. Afterward, accounting for the impact of classification success on neighboring pixels within SAR images for each band, the weight of the affected neighborhood pixels is computed. These two weights vary, and the final weight is taken after normalization. The mean of the weights of the BPAs from various bands is employed to generate a mean BPA, which is then merged to produce the ultimate classification result.

## 7. Discussion and Conclusions

Presenting and analyzing the above five papers, we can confidently conclude that the proposed decision fusion techniques exhibit diverse strengths and limitations in enhancing classification accuracy and addressing challenges in urban and land cover mapping applications. The two-step decision fusion strategy presented in [18] showcases improved classification accuracy by leveraging the complementary strengths of hyperspectral and multispectral images while addressing spatial and semantic uncertainties. However, it introduces complexities in computational processes and data integration. The framework based on Markov and conditional random fields in [24] employs multiple decision sources to enhance classification accuracy, yet it may pose challenges in computational complexity and demand more extensive training data. The fusion of MODIS and Landsat data in [48] proves promising in improving overall accuracy but faces challenges in the classification accuracy of MODIS data and preconditions for linear fusion models. The integration of optical and SAR images [77] demonstrates improved accuracy and efficient computation but is sensitive to speckle and demands higher memory occupation. Lastly, the decision-level combination of multi-band information in SAR image fusion [71] presents enhanced classification accuracy but introduces computational complexity and longer processing times. These findings underscore the need for careful consideration of trade-offs and potential challenges in adopting decision fusion techniques for specific applications.

The exploration of decision fusion techniques in land cover classification, particularly with remote sensing imagery and multisource data, reveals significant advancements and potential challenges. The reviewed papers underscore the effectiveness of various decision fusion methods, such as global regularization optimization, innovative hyperspectral classification methodologies, three-layer decision fusion and convolution-based computations for addressing the minimum energy problem. These approaches contribute to improving pixel-level classification accuracy and robustness in geospatial analysis.

Specifically, the methodologies and techniques discussed in this review paper offer significant contributions with wide-ranging applications across various domains. The fusion of decision-making algorithms with hyperspectral–multispectral data, as highlighted by the works [18,24] and with optical–SAR fusion [77], presents opportunities for enhanced precision in applications such as precision agriculture, environmental monitoring, urban planning, disaster management and infrastructure inspection. For instance, in precision agriculture, farmers can utilize hyperspectral imaging combined with classification techniques to monitor crop health, detect early signs of diseases and optimize irrigation strategies. Similarly, environmental agencies can leverage these techniques to assess the impact of human activities on natural landscapes, for example, by monitoring changes in forest cover or wetland ecosystems. The integration of MODIS and Landsat data, as explored in [48], underscores the potential for improved land cover classification, benefiting sectors like agricultural planning, disaster management and biodiversity conservation. For example, agricultural planners can use the classified land cover information to optimize crop selection and water resource management, while conservationists can identify critical habitats and ecological corridors for targeted conservation efforts. Furthermore, the utilization of SAR image fusion classification, as delineated in [71], demonstrates promising avenues in environmental monitoring, disaster management, infrastructure planning and security applications. Emergency response agencies can operationalize these techniques for rapid assessment of land cover changes in the aftermath of natural disasters, facilitating more efficient disaster response and recovery efforts. Collectively, these advancements not only underscore the robustness of the methodologies but also emphasize their practical relevance and potential impact for end users across diverse sectors.

Beyond the key findings, the broader landscape of decision fusion techniques in urban and land cover mapping presents a diverse set of strengths, challenges and future opportunities. The two-step decision fusion strategy demonstrates promise for enhanced accuracy, with prospects for integrating additional data sources like LiDAR or radar. Frameworks based on Markov and conditional random fields offer avenues for parameter learning and addressing challenges related to limited training data. Challenges identified in the fusion of MODIS and Landsat data, as well as optical–SAR decision fusion, underscore the need for continuous improvement in handling mixed pixel problems and spatial resolution challenges and embracing emerging technologies.

Looking forward, future opportunities include the integration of more data sources, exploration of deep learning approaches, addressing computational complexity and heterogeneity in SAR data, uncertainty quantification and operational implementation. The development of user-friendly tools, advanced machine learning techniques, real-time processing and interdisciplinary collaborations is crucial for the evolution of decision fusion methodologies in remote sensing and geospatial analysis. The collective insights from these studies contribute to the expanding body of knowledge in land cover classification, paving the way for continuous advancements in the robustness of geospatial analysis tasks.

## Data Availability

Data are available upon request.
